# Ossification of Bilateral Sacrotuberous Ligaments: Two Cases Report and Literature Review

**DOI:** 10.1111/os.14062

**Published:** 2024-04-14

**Authors:** Ruzai Chu, Qiaoyuan Jiang, Shijun Chai, Zhengbao Pang, Yifan Xu, Xing Zhao

**Affiliations:** ^1^ Department of Orthopaedic Surgery, Sir Run Run Shaw Hospital Zhejiang University School of Medicine Hangzhou China; ^2^ Department of Orthopaedic Surgery The People's Hospital of Tiantai County Taizhou China; ^3^ School of Medicine Wenzhou Medical University Wenzhou China

**Keywords:** Ossification, Pathogenesis, Pudendal nerve entrapment, Sacrotuberous ligament

## Abstract

Ossification of the sacrotuberous ligament is a rare occurrence in soft tissue, with only 15 cases reported in the past few decades. We reported two cases of bilateral ossification in sacrotuberous ligaments and provided a concise review of the literature on this pathology. Clinical data, radiographic outcomes, and diagnostic and treatment details were obtained. This study aimed to summarize this disease's characteristics and investigate its pathogenesis through a review of literature from the last thirty years. This condition is often incidentally confirmed in elderly males *via* imagiological examination or gross anatomy and presents a low morbidity rate. Its pathogenesis may be related to stress concentration, excessive intake of element ions, injury repair, and improper operative technique. The majority of patients may not exhibit any clinical symptoms or signs and typically do not require medical interventions. It may be complicated with pudendal nerve entrapment syndrome. The long‐term effects of surgical resection and the most effective treatment approach remain areas for further research.

## Graphical abstract

This is an exceptionally rare ossification of the ligament, which appears to only affect male patients. The pathological tissue takes on the shape of an antelope horn, leaving a lasting impression on observers.

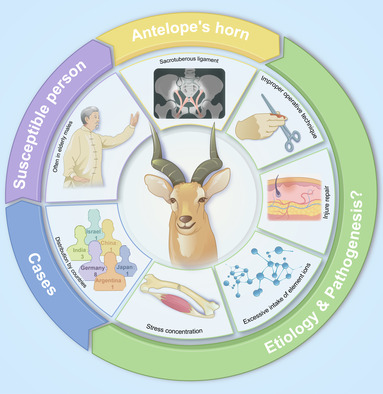

## Introduction

The sacrotuberous ligament (STL) originates from the posterior superior iliac spine, the lower part of the sacrum, and the upper part of the lateral margin of the coccyx, extending obliquely downward and outward. It crosses behind the sacrospinous ligament and terminates at the medial margin of the ischial tuberosity.^1^, ^2^ The STL is critical in stabilizing the pelvis, which is a crucial structure for maintaining the stability of the pelvic ring.^3^, ^4^, ^5^, ^6^

Ossification of soft tissue, specifically in STL, is an exceptionally rare occurrence. The first case was reported by Gruber in 1876, with bilateral ossification in an elderly male.^7^ Because the STL is a crucial structure in maintaining pelvic stability, its ossification or dysfunction may be a rare and even undiscovered cause of pain or discomfort in the lumbosacral, perineal, and buttock regions. This poses certain challenges in our clinical practice and could potentially serve as a new direction or topic for research among orthopedic surgeons. The current cases were reported and reviewed in terms of the general characteristics, epidemiology, etiology, pathogenesis, clinical manifestations, diagnosis, differential diagnosis, and therapeutic strategies of the disease.

## Case Presentation

### Case 1

A 70‐year‐old man presented to our clinic, reporting pain in his right hip following a fall. He recalled a similar incident occurring approximately two years prior, where he landed on his buttocks, but did not seek any medical treatment at the time. The patient described experiencing localized pain and discomfort in his hips, exacerbated by prolonged sitting or squatting. During the physical examination, we observed significant tenderness in the bilateral ischial tuberosity regions. Subsequent tests were conducted, yielding the following results.

**FIGURE 1:**
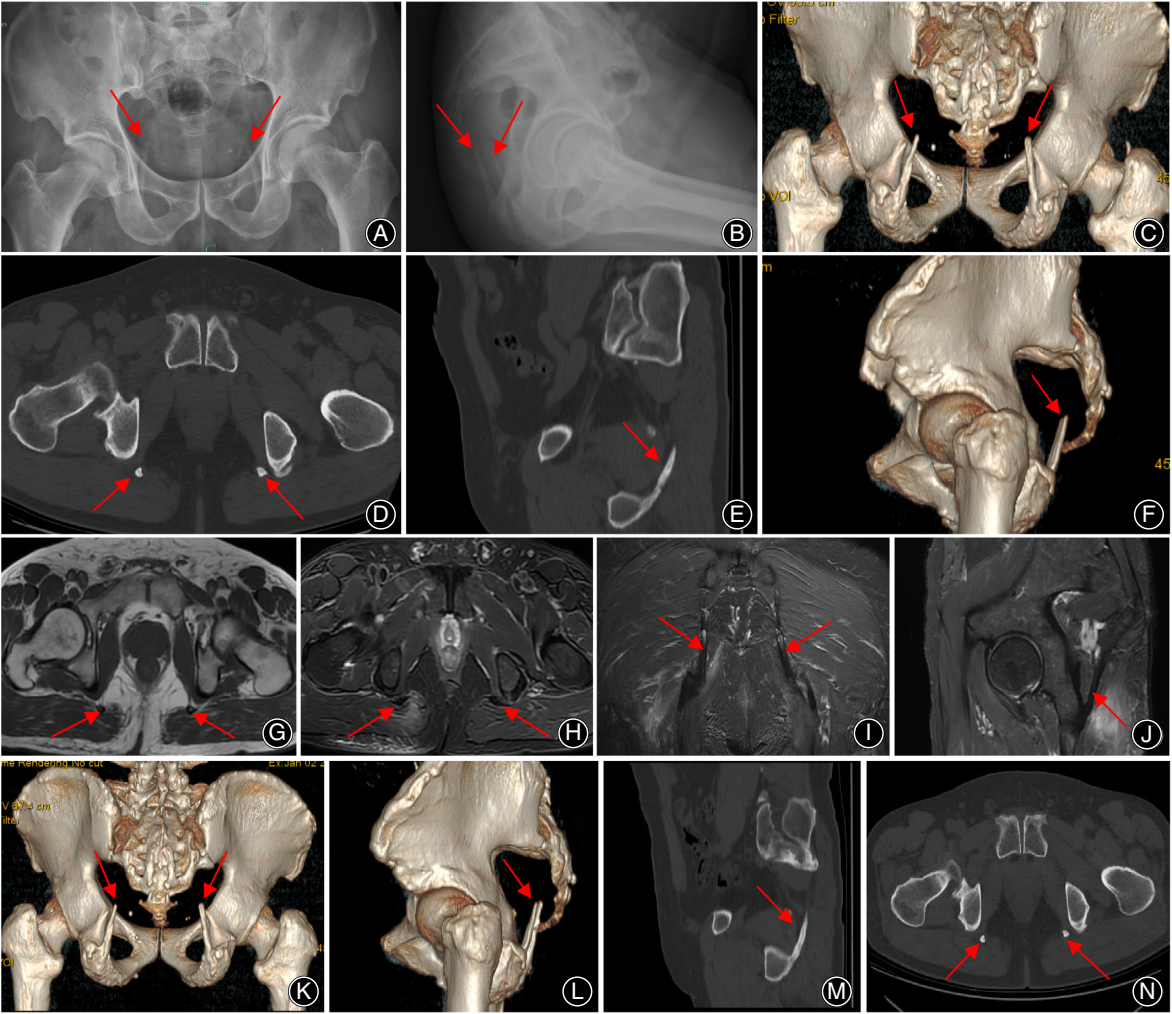
X‐ray, CT, and MRI images of the patient. (A, B) “Antelope's horns” sign on anteroposterior and lateral views of pelvis on x‐ray imaging. (C–F) CT showed a shape resembling fish bones. (G–J) MRI revealed on oblique sagittal (15 degrees) and cross‐sectional views of T1 and T2. (K–N) The follow‐up CT images taken after 12 months indicated that there were no noticeable changes in the ossification areas.

The anteroposterior and lateral views of the pelvis on X‐ray imaging revealed symmetrical strips of high‐density shadows following the sacrotuberous ligaments (STLs), each with a smooth, broad‐based edge originating from the bilateral ischial tuberosities. These strips, resembling antelope horns, measured approximately 6.8 centimeters on the left and 6.9 centimeters on the right. Three‐dimensional computed tomography (CT) scanning of the pelvis showed a structure akin to fish bones. These symmetrically connected structures extended from the bilateral ischial tuberosities, featuring a uniform density that gradually tapered from distal‐lateral to proximal‐medial regions. The magnetic resonance imaging (MRI) of the STLs was assessed using oblique sagittal (15 degrees) and cross‐sectional views with T1 and T2‐weighted sequences. The images revealed a belt‐shaped area of hypointense signal, accompanied by a platy‐shaped area of hyperintense signal in the middle on the T1‐weighted sequence. Additionally, the T2‐weighted sequence displayed edema signal in the soft tissue at the rear of the right STL (Figure 1). Laboratory tests were conducted, including blood routines, blood biochemistry, rheumatoid factor, antistreptolysin O test, high sensitivity C‐reactive protein, erythrocyte sedimentation rate, and human leukocyte antigen B27. The results of these tests showed no abnormalities. The clinical diagnosis for the patient was ossification of the bilateral STLs.

The patient was prescribed celecoxib capsules, to be taken twice daily for a duration of two weeks. In addition, he was advised to apply a towel, warmed to approximately 45 degrees Celsius, to his hips once per day for a week. He was also guided to engage in a targeted exercise regimen aimed at strengthening his biceps femoris muscles. This regimen included exercise such as hamstring curls. Follow‐up visits at two weeks, three months, six months, and twelve months revealed a gradual alleviation of the patient's symptoms. However, the tenderness in his hips persisted, and radiological imaging indicated no significant changes in the ossified areas.

### Case 2

**FIGURE 2:**
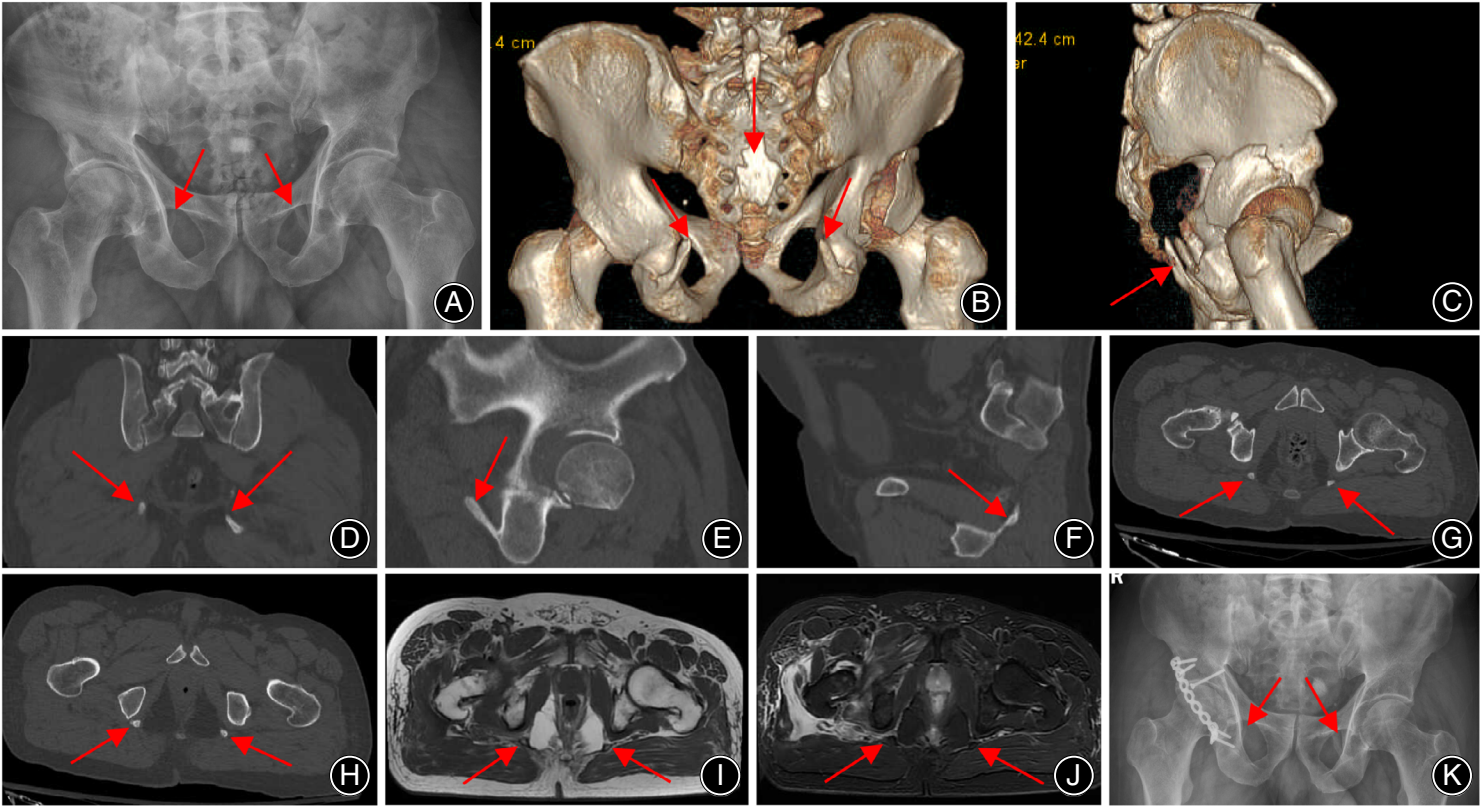
The patient's imaging data. (A) Anteroposterior view of pelvis on X‐ray displayed the injury of right hip joint and ossification of bilateral sacrotuberous ligaments. (B–H) CT scanning revealed typical signs of ossified sacrotuberous ligaments, with ossified signal observed at the attachment of the sacrum in three‐dimensional reconstruction. (I, J) Cross‐sectional views of T1 and T2 sequences of MRI showed the bilateral sacrotuberous ligaments. (K) Postoperative x‐ray image.

This case involved a 64‐year‐old man with no previous history of hip injury or pain. Before his admission to the hospital following a traffic accident, he was in good overall health. The physical examination revealed that his right hip exhibited deformity due to flexion, adduction, and pronation, accompanied by noticeable elastic fixation and tenderness. Laboratory tests were within normal ranges. Radiological evaluations, including X‐ray, CT, and MRI, identified a fracture in the right acetabulum and dislocation of the femoral head. The imaging also indicated calcification in the bilateral STLs (Figure 2). The patient received open reduction and internal fixation for the acetabular fracture and femoral head dislocation. Postoperative care included skin traction and initiating early rehabilitation exercises. The ossification of the STLs was not specifically treated.

## Discussion

### General Characteristics and Epidemiology

After reviewing the literature on ossification of STL from 1993 to 2022, we collated all related cases (Table 1). In this summary, four cases were identified through clinical findings, and 11 were detected in gross anatomy, with all instances occurring in males. Of these 15 cases, seven were bilateral, and eight were unilateral. Age data were available for 12 cases, while three cases lacked age information. The reported instances originated from different countries.

**TABLE 1:** Summary of the cases of ossification of sacrotuberous ligament

No./study	Sex/age	PT	Side	Occupation	Symptom/sign	Treatment
Prescher and Bohndorf^8^ 1993	M/81	GER	B	Shopkeeper	NA/NA	NA
	M/81	GER	B	Miner	NA/NA	NA
	M/84	GER	B	Waiter	NA/NA	NA
	M/79	GER	B	Miner	NA/NA	NA
	M/69	GER	U(R)	Painter	NA/NA	NA
	M/76	GER	U(L)	Butcher	NA/NA	NA
	M/81	GER	B	Miner	NA/NA	NA
	M/47	GER	U(L)	Homeless	NA/NA	NA
Zhou *et al*.^9^ 2000	M/79	CHN	B	NA	Pain in hips when sitting /NA	Take NSAIDs
Shaul *et al*.^10^ 2002	M/42	ISR	U(R)	Driver	Pain and discomfort in right hip when sitting /NA	Take NSAIDs
Arora *et al*.^6^ 2009	M/NA	IND	U(R)	NA	NA/NA	NA
Sandri *et al*.^11^ 2013	M/77	JPN	U(R)	NA	Severe pain and tenderness in right hip with PNE syndrome	Surgical excision
Saal *et al*.^12^ 2016	M/71	ARG	B	NA	Lower back pain /NA	NA
Tirpude and Ravi^13^ 2018	M/NA	IND	U(L)	NA	NA/NA	NA
Yuvaraj *et al*.^14^ 2018	M/NA	IND	U(R)	NA	NA/NA	NA
This study (*case 1*) 2022	M/70	CHN	B	Officer	Localized pain and tenderness in hips after prolonged sitting	Take NSAIDs; Physiotherapy
This study (*case 2*) 2023	M/64	CHN	B	Carpenter	No clinical manifestations	Without any treatment

Abbreviations: ARG, Argentina; B, bilateral; CHN, China; GER, Germany; IND, India; ISR, Israel; JPN, Japan; L, left; M, male; NA, not available; NSAIDs, non‐steroidal anti‐inflammatory drugs; PNE, pudendal nerve entrapment; PT, publication territory; R, right; U, unilateral.

According to our research, ossification of STL is a rare occurrence involving calcification in soft tissue or heterotopic ossification (HO). Previous reports have not documented any cases in female or juvenile. Typically, it is confirmed through imageological examination in clinical practice or *via* gross anatomical studies in the laboratory, predominantly occurring in elderly men with a low incidence rate. This condition generally manifests after the age of 65, suggesting a potential degenerative change in the pelvic region. There is some variability in findings concerning unilateral *versus* bilateral occurrences. While our study observed no significant difference, Yuvaraj *et al*. reported a less frequent incidence of bilateral cases.^14^

The ossified ligament is commonly described as having the shape of a slate‐pencil, goat's horn or violin string.^6^, ^9^ It has a broad‐based origin and gradually tapered from distal‐lateral to proximal‐medial end. The terminal part may be acicular or fasciculate, and the abnormal ligament can vary in length between 1.1 and 7.2 cm, as observed in the study by Prescher and Bohndorf.^8^ In their research, which involved the examination of 101 cadavers, eight cases of ossification were identified. They have concluded that while this pathology is not a reliable indicator of spinal diffuse idiopathic skeletal hyperostosis (DISH), it is an acquired lesion potentially linked to injury of the STL. In contrast to other study, Haller *et al*. believed that the typical sign of spinal ligamentous ossification in DISH could also occur in extraspinal locations.^15^

### Etiology and Pathogenesis

After collecting the occupational data for all cases, it becomes clear that many of the occupations necessitate prolonged sitting, stooping, or hip flexion. This pattern suggests a possible link between the etiology of the condition and specific occupational demands, which may stem from the concentration of stress and local tissue hypoxia due to maintaining a posture for extended durations.

Zhou *et al*. documented a case of bilateral lesions, diagnosed concurrently as skeletal fluorosis *via* X‐ray testing in 2000, attributed to excessive fluoride intake.^9^ They elaborated that the characteristic “goat's horns” sign observed in X‐ray imaging could also manifest in conditions like ankylosing spondylitis and cadmium poisoning. It is hypothesized that excessive fluoride consumption may influence the body's calcium ion distribution, potentially leading to the calcification of soft tissue including ligaments, muscles, and teeth.

For decades, post‐traumatic HO has been regarded as an uncommon complication following central nervous system trauma or an intractable side effect of major surgical procedures.^16^, ^17^ However, existing literature indicates that HO in peripheral tissue is relatively frequent after orthopedic surgeries, severe burns, brain or spinal cord injuries, and blast injuries encountered in combat.^16^, ^18^, ^19^, ^20^ Nonetheless, post‐traumatic or post‐operative calcification of the STL remains a rare occurrence. Shaul *et al*. reported a case illustrating this phenomenon, where calcification was described as a consequence of inflammatory repair and vascular degeneration. Their study included a 42‐year‐old male taxi driver who experienced ossification of the right STL, leading to hip pain and discomfort after prolonged sitting. This patient had previously undergone an open reduction and internal fixation for a pelvic injury about six months prior.^10^ Factors contributing to post‐operative HO may include the extent of soft tissue dissection, the presence of residual bone fragments, and hematoma.^18^

### Clinical Manifestations and Diagnosis

The majority of patients with ossification of STL exhibit no clinical symptoms or signs. The presence and severity of symptoms or signs are influenced by the size and length of the ossified tissue, and the degree of damage or pressure it places on adjacent tissue. Clinical manifestations often include hip pain, discomfort, and palpable tenderness. Unlike HO in other regions, ossification of the STL rarely affects limb and joint mobility. It has been suggested that ossification of the STL could potentially lead to PNE syndrome, causing chronic perineal pain.^6^, ^11^, ^14^, ^21^ The definitive diagnostic tools for HO are CT and X‐ray.^19^, ^20^

### Differential Diagnosis

It is crucial to distinguish hip pain or discomfort caused by other conditions, such as sciatica, lumbar disc degeneration, piriformis syndrome, sacroiliitis, ischiogluteal bursitis, STL injury, hematoma, and bone tumor. Notably, ischiogluteal bursitis, once known as “weaver's bottom,” shares similar clinical features. However, in ischiogluteal bursitis, a mass at the ischial tubercle is often palpable, and X‐ray or CT scanning typically shows no positive findings. Therefore, further diagnostic procedures like MRI and B‐ultrasound are often necessary for accurate diagnosis.^22^, ^23^

### Therapeutic Strategies

Therapeutic strategies for ossification of the STL have been reported. The NSAIDs may be prescribed to alleviate symptom such as pain. While radiotherapy and bisphosphonates might be effective in preventing HO, current clinical practice lacks sufficient experience and evidence to substantiate this claim.^16^, ^17^, ^20^ The STL is considered as a continuation of the origin of the long head of biceps femoris.^5^, ^14^, ^24^ Patients with STL pain can often experience benefits through the injection of local anesthetic, relaxation of biceps femoris, and gluteus maximus contraction training.^25^, ^26^ Surgical resection was a choice for some cases.^19^, ^20^ Sandri *et al*. described a challenging case of intractable PNE syndrome, where the patient underwent surgical removal of the lesion due to intense and persistent pain in the right ischial tubercle and pudendal area.^11^ It is hypothesized that surgical resection might risk damaging the ligament, potentially leading to pelvic instability. This instability could, in turn, result in secondary pain or movement disorders. The ossification of STL could potentially be an uncommon etiological factor contributing to PNE syndrome. Historically, treatment approaches for PNE syndrome have varied, spanning from conservative measures such as lifestyle modifications, analgesics, and pelvic floor physiotherapy to invasive measures such as anesthetic pudendal nerve block, botulinum toxin injections, surgical decompression, radiofrequency, and neuromodulation therapies. But there has never been a consensus on the diagnosis and treatment of PNE syndrome, which often brings us great challenges in clinical practice. The pulsed radiofrequency technique, known for its minimally invasive, safe, and precise nature, has shown promise in treating PNE syndrome.^27^ Therefore, it may be a preferable option for treating the case of ossification of the STL with PNE syndrome.

## Conclusion

The majority of patients with ossification of STL may not require any medical interventions. For those who experience pain or discomfort may achieve positive outcomes through conservative treatment and functional exercise. Furthermore, these patients can maintain a stable state for a long time. Whether surgical resection produces long‐term complications and what is the most appropriate treatment need further study.

## Ethics Statement

The present study was approved by the Ethics Committee of the People's Hospital of Tiantai County (2023 Research No. 020).

## Author Contributions

Ruzai Chu and Qiaoyuan Jiang contributed to the conception and design of the study, acquisition and analysis of data, interpretation of data and drafting of the article. Xing Zhao, Shijun Chai, Zhengbao Pang and Yifan Xu reviewed and edited the manuscript for important intellectual content and provided critical revisions. The literature searches were performed by all authors. Finally, the first draft of the manuscript was written by Ruzai Chu and Qiaoyuan Jiang. All authors read and approved the final manuscript.

## Funding Information

No funds were received in support of this work. No benefits in any form have been or will be received from a commercial party related directly or indirectly to the subject of this manuscript.

## References

[1] Standring S , Ellis H , Healy J , Johnson D , Williams A . Gray's anatomy. 39th ed. London: Elsevier Churchill Livingstone; 2005. p. 1428–1439.

[2] Simmons DN . Sonography of the sacrotuberous ligament. ASUM Ultrasound Bull. 2008;11(2):25–30.

[3] Vleeming A , Stoeckart R , Snijders CJ . The sacrotuberous ligament: a conceptual approach to its dynamic role in stabilising the sacroiliac joint. Clin Biomech. 1989;4(4):201–203.

[4] Williams PL , Bannister LH , Berry MM . Gray's anatomy: the anatomical basis of medicine and surgery. 38th ed. Edinburgh, Scotland: Churchill Livingstone; 1995. p. 677–879.

[5] Woodley SJ , Kennedy E , Mercer SR . Anatomy in practice: the sacrotuberous ligament. NZ J Physiother. 2005;33(3):91–94.

[6] Arora J , Mehta V , Suri RK , Rath G . Unilateral partial ossification of sacrotuberous ligament: anatomico‐radiological evaluation and clinical implications. Rom J Morphol Embryol. 2009;50(3):505–508. 19690783

[7] Gruber W . Anatomische notizen. Virchows Arch. 1876;66:447–454.

[8] Prescher A , Bohndorf K . Anatomical and radiological observations concerning ossification of the sacrotuberous ligament: is there a relation to spinal diffuse idiopathic skeletal hyperostosis (DISH)? Skeletal Radiol. 1993;22(8):581–585. 8291010 10.1007/BF00197139

[9] Zhou Q , Zhao D , Zhang M . Ossification in bilateral sacrotuberous ligament caused by fluorosis: a report of one case. J Appl Radiol. 2000;10:625 [In Chinese, English title.].

[10] Shaul B , Meir L , Rami M . Myositis ossificans circumscripta of the sacrotuberous ligament: a case report and review of the literature. J Orthop Trauma. 2002;16(9):672–674. 12368649 10.1097/00005131-200210000-00010

[11] Sandri A , Regis D , Toso M , Bartolozzi P . Surgical removal of a partial ossified sacrotuberous ligament for refractory pudendal nerve entrapment syndrome. J Orthop Sci. 2013;18(4):671–674. 22427016 10.1007/s00776-012-0202-3

[12] Saal A , Abraham CR , Dalmasso MV . Bilateral calcification of sacrotuberous ligament. Medicina (B Aires). 2016;76(5):316. 27723621

[13] Tirpude A , Ravi P . Partial ossification of left sacrotuberous ligament and bilateral ossification of transverse acetabular ligament with sacralisation of left part of l5 vertebra: a case report. J Anat Soc India. 2018;67(1):S15.

[14] Yuvaraj M , Priyadarshini A , Rajkumar S . Ossified sacrotuberous ligament and its clinical significance: a case report. Int J Pediatr. 2018;6(1):6981–6985.

[15] Haller J , Resnick D , Miller CW , Schils JP , Kerr R , Bielecki D , et al. Diffuse idiopathic skeletal hyperostosis: diagnostic significance of radiographic abnormalities of the pelvis. Radiology. 1989;172(3):835–839. 2788894 10.1148/radiology.172.3.2788894

[16] Eisenstein N , Stapley S , Grover L . Post‐traumatic heterotopic ossification: an old problem in need of new solutions. J Orthop Res. 2018;36(4):1061–1068. 29193256 10.1002/jor.23808

[17] Osorio T , Lemos J , Tkachuk O , Sousa R , Vasques A . Surgical resection combined with adjuvant radiotherapy and non‐steroidal anti‐inflammatory drugs in the treatment of heterotopic ossification following total hip arthroplasty. Acta Med Port. 2023;36(3):202–205. 35748410 10.20344/amp.18230

[18] Vanden Berge D , Bondar K , Yakkanti R , Constantinescu D , Carvajal Alba JA . Severe heterotopic ossification after revision total knee arthroplasty: a case report and review of the literature. J Am Acad Orthop Surg Glob Res Rev. 2022;6(11):e22.00053. 10.5435/JAAOSGlobal-D-22-00053 PMC9663138 36733984

[19] Mujtaba B , Taher A , Fiala MJ , Nassar S , Madewell JE , Hanfy AK , et al. Heterotopic ossification: radiological and pathological review. Radiol Oncol. 2019;53(3):275–284. 31553710 10.2478/raon-2019-0039 PMC6765162

[20] Cholok D , Chung MT , Ranganathan K , Ucer S , Day D , Davis TA , et al. Heterotopic ossification and the elucidation of pathologic differentiation. Bone. 2018;109:12–21. 28987285 10.1016/j.bone.2017.09.019 PMC6585944

[21] Loukas M , Louis RG , Hallner B , Gupta AA , White D . Anatomical and surgical considerations of the sacrotuberous ligament and its relevance in pudendal nerve entrapment syndrome. Surg Radiol Anat. 2006;28(2):163–169. 16463079 10.1007/s00276-006-0082-3

[22] Roh YH , Yoo SJ , Choi YH , Yang HC , Nam KW . Effects of inflammatory disease on clinical progression and treatment of ischiogluteal bursitis: a retrospective observational study. Malays Orthop J. 2020;14(3):32–41. 33403060 10.5704/MOJ.2011.007 PMC7752025

[23] Cho KH , Lee SM , Lee YH , Suh KJ , Kim SM , Shin MJ , et al. Non‐infectious ischiogluteal bursitis: MRI findings. Korean J Radiol. 2004;5(4):280–286. 15637479 10.3348/kjr.2004.5.4.280 PMC2698173

[24] Bierry G , Simeone FJ , Borg‐Stein JP , Clavert P , Palmer WE . Sacrotuberous ligament: relationship to normal, torn, and retracted hamstring tendons on MR images. Radiology. 2014;271(1):162–171. 24475819 10.1148/radiol.13130702

[25] Sasaki T , Kurosawa D , Murakami E , Watanabe T . Physical therapeutic options for residual sacrotuberous ligament pain after treatment of sacroiliac joint dysfunction. J Phys Ther Sci. 2021;33(9):646–652. 34539068 10.1589/jpts.33.646 PMC8436043

[26] Sasaki T , Kurosawa D , Murakami E . Sacrotuberous ligament pain in patients who underwent sacroiliac joint arthrodesis: incidence and management of post‐surgical lower‐buttock pain. Spine Surg Relat Res. 2022;6(5):555–562. 36348684 10.22603/ssrr.2021-0239 PMC9605753

[27] Luesma MJ , Gale I , Fernando J . Diagnostic and therapeutic algorithm for pudendal nerve entrapment syndrome. Med Clin (Barcelona). 2021;157(2):71–78. 10.1016/j.medcli.2021.02.012 33836860

